# Beta Caryophyllene-Loaded Nanostructured Lipid Carriers for Topical Management of Skin Disorders: Statistical Optimization, In Vitro and Dermatokinetic Evaluation

**DOI:** 10.3390/gels9070550

**Published:** 2023-07-06

**Authors:** Mohammed Ghazwani, Umme Hani, Mohammed H. Alqarni, Aftab Alam

**Affiliations:** 1 Department of Pharmaceutics, College of Pharmacy, King Khalid University, Abha 61441, Saudi Arabia; uahmed@kku.edu.sa; 2 Department of Pharmacognosy, College of Pharmacy, Prince Sattam Bin Abdulaziz University, Al Kharj 11942, Saudi Arabia; m.alqarni@psau.edu.sa (M.H.A.); a.alam@psau.edu.sa (A.A.)

**Keywords:** β-caryophyllene, nanostructured lipid carriers, skin disorders, heat emulsification, traditional gel formulation

## Abstract

This work aimed to overcome the disadvantages of the oral administration of beta-caryophyllene and boost efficiency by developing a nanostructured lipid carrier for topical administration of the drug in skin disorders. The heat emulsification method was utilized to produce beta-caryophyllene-loaded nanostructured lipid carriers. The newly created formulation was examined for its particle size, entrapment efficiency, and zeta potential after being improved using the Box–Behnken Design. The chosen formulation underwent tests to determine its ex vivo skin retention, dermatokinetic, in vitro release, antioxidant, and confocal laser scanning microscopy study. The findings of the characterization of the nanostructured lipid carriers demonstrated that the particles had a spherical form and a size of 210.86 nm (0.263 polydispersity index). The entrapment efficiency was determined to be 86.74%, and the zeta potential was measured to be −26.97 mV. The in vitro release investigation showed that nanostructure lipid carriers were capable of releasing regulated amounts of beta-caryophyllene for up to 24 hrs. In comparison to the traditional gel formulation, the ex vivo investigation demonstrated a 1.94-fold increase in the skin’s capacity to retain the substance. According to the findings of the study, nanostructure lipid carriers loaded with beta-caryophyllene have the potential to be investigated for use as a topical administration method in skin disorders with enhanced skin retention and effectiveness.

## 1. Introduction

β-caryophyllene (BCP) is a chemical observed in plants and is classified as a bicyclic sesquiterpene. It is most commonly found in nature in the form of trans-caryophyllene, which may be found in combination with the oxidized product β-caryophyllene oxide as well as the trace quantity of its isomers (Z)-iso-caryophyllene and α-caryophyllene [1]. The main active component of essential oil is BCP, which is generated from a wide variety of plants used in the culinary and spice industries. According to the Essential Oil Database, BCP is a volatile phyto-compound that is obtained from cinnamon, cloves, cannabis, lavender, black pepper, oregano, basil, and rosemary. Anti-inflammatory [2], anti-carcinogenic [3], anti-bacterial [4], anti-oxidative [5], and analgesic [6] properties are amongst its reported biological effects. BCP is classified as a cannabinoid (CB): more specifically, a phyto-cannabinoid (pCB). Cannabinoids, both natural and synthetic, have the capacity to stimulate activity in cannabinoid receptors (CB1 and CB2) [7]. Because it is recognized to particularly bind to the CB2 receptor, beta-caryophyllene is occasionally also categorized as an atypical cannabinoid [8]. This is because of how the CB2 receptor interacts with it. CB1 is the receptor that is accountable for the psycho-active effects that are linked with specific cannabinoids such as tetrahydrocannabinol (THC). The therapeutic target is a CB2 for the action of osteoporosis, inflammation, and atherosclerosis [9], mainly in the peripheral tissues of the body. It has been demonstrated that beta-caryophyllene is directly advantageous for a variety of conditions and disease types, including colitis [10], osteoarthritis [11], diabetes [12], anxiety and depression [13], liver fibrosis [14], and Alzheimer’s [15]. In cancer studies, beta-caryophyllene is reported to exhibit synergism with the chemotherapeutic agent paclitaxel on human tumor cell lines. Even when used on its own, the compound promoted apoptosis and inhibited the development of tumors. Using Caenorhabditis elegans as a model, researchers found that beta-caryophyllene might alter genes associated with stress and inherits a potential to increase the organism’s lifespan [16].

Among its many biological functions, BCP has an anti-inflammatory effect by inhibiting the main inflammatory mediators, such as nuclear factor kappa-light chain-enhancer of activated B cells (NF-B), cyclooxygenase 1 (COX-1), and cyclooxygenase 2 (COX-2). Numerous IVIV (in vitro and in vivo) studies suggested that the therapy with BCP betters the outcomes in nervous system diseases, atherosclerosis, and tumors [17,18,19,20,21,22]. In addition, pre-clinical research has shown that BCP may be beneficial in the treatment of Streptococcus infections, osteoporosis, and steatohepatitis; it also possesses anti-convulsant, analgesic, myorelaxant, sedative, and antidepressant properties. BCP is not hazardous to rats, since the dose required to cause 50% mortality (LD50) is more than 5000 mg/kg. Despite this, it inhibits a number of different cytochromes P450 (CYP450) isoforms, most notably CYP3A4, which are responsible for the metabolism of xenobiotics. This might result in undesirable consequences owing to drug levels that are over the therapeutic window [23].

This research project’s goal was to develop and evaluate a BCP-loaded nanostructured lipid carrier (BCP-NLC) for the management of topical skin disorders. This report also focuses on boosting permeability through the skin bioavailability of BCP by avoiding any barrier. The ability of the BCP-NLC to alleviate skin disorder was additionally examined by performing in vitro and ex vivo permeation studies as well as dermatokinetics, antioxidant, and confocal laser scanning microscopy (CLSM) studies.

## 2. Results and Discussion

### 2.1. Precursory Examining of Solid Lipids, Liquid Lipids and Surfactant

**Figure 1:**
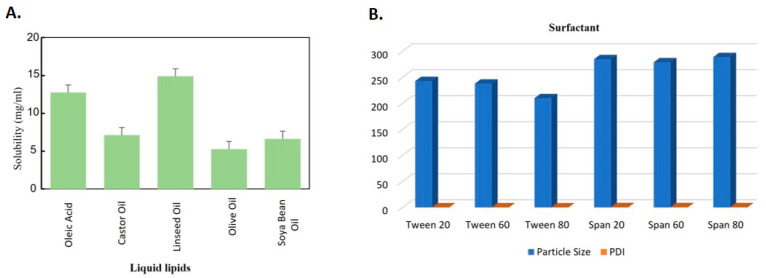
Solubilization of BCP in various; (**A**) Liquid lipids, (**B**) Surfactants.

Supplementary Figure S1 and Supplementary Table S1 describe the results of the experiment that found the solubility of BCP in solid lipids to be the greatest in Compritol 888ATO (*n* = 3). Figure 1 and Supplementary Table S2 display the findings of the investigation on the degree to which BCP was soluble in liquid lipids (*n* = 3). The greatest degree of solubility was demonstrated by linseed acid. BCP was tested in various lipids until saturation solubility; it was determined that Compritol and linseed acid exhibited better solubility capability for BCP in comparison to the selected lipids. As per the evaluation, only two lipids were taken for this study. Figure 1 and Supplementary Table S3 display the results of the selection of surfactant that was made according to particle size and Polydispersity Index (PDI) (*n* = 3). It was found that Tween 80 provided the greatest degree of solubility for BCP. As a result, Tween 80 was utilized further in the process of NLC production. The data are statistically significant with a *p*-value less than 0.005.

### 2.2. Experimental Design

The design of expert methodology was utilized so that we could investigate how different formulation and process factors impacted the resulting formulation’s desired properties. Utilizing design-expert software, a Box–Behnken Design (BBD) design was carried out with 17 trials, each consisting of three factors and two levels. The material properties, lipid and surfactant concentration were chosen, and the sonication duration was chosen as the process parameter. As essential quality aspects of the suggested formulation, the particle size and entrapment efficiency (EE) were chosen as dependent variables to study. For the purpose of optimizing the formulation, the quantity of lipid, surfactant, and the sonication period were each investigated at three different levels: low, medium, and high. Supplementary Table S4 of the supplemental materials provides a summary of the 17 batches that were produced after the concept was put into action. The tests were carried out by changing the value of the independent variables while maintaining the values of all other factors. Particle size and EE were two response factors and dependent variables that were explored. Supplementary Table S4 of the supplemental materials provides a summary of the data.

### 2.3. Independent Variables Effect on the Particle Size

**Figure 2:**
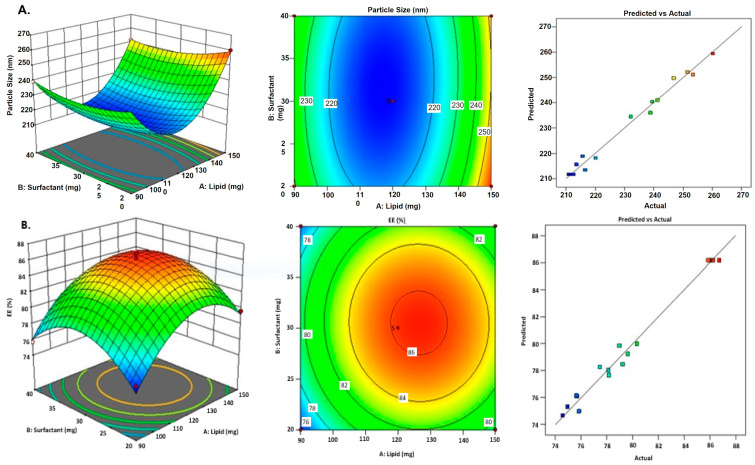
Representation of 3D surface contour plot; (**A**) Predicted vs. actual on the particle size, (**B**) Predicted vs. Actual on the entrapment efficiency.

As per Supplementary Table S4, it was discovered that the particle size of the BCP-NLC dispersion fell somewhere in the range of 210.86 to 260.23 nm. The relevance of the model may be deduced from the fact that its quadratic model has an F-value of 64.15. In Figure 2A, we see the 3D plot, the contour plot, and a comparison of the anticipated value of the response variable particle size to the actual value. With a 43.05 F-value in comparison to the pure error, it was determined that the Lack of Fit was a significant finding (*p* < 0.0001). The values that were coded for the independent variables that were chosen are shown as an equation here.

Y1 = +211.68 + 7.55A − 2.01B − 0.7163C − 1.69AB + 0.0350AC − 0.3625BC + 31.42A^2^+ 5.07B^2^ − 0.2840C^2^

where A—Lipid concn. (in mg); B—Surfactant concn. (in mg); C—Sonication time (in minute).

The R^2^ value (0.9880), the adjusted R^2^ value (0.9726), and the predicted R^2^ value (0.8135) were discovered to be in near agreement with one another (the variance between them was lower than 0.2). The absence of a fit demonstrates that the data of the best-fitting model are comparable, which validates the accuracy of the model’s ability to account for experimental findings. The upsurge in particle size that occurs in response to an upsurge in lipid concentration is represented by the regression equation presented above. Increases in both the concentration of the surfactant and the length of time spent on sonication led to a smaller reduction in the vesicle size. With a rise in the total amount of lipid, the viscosity of the dispersed phase, also known as the melted lipid phase, will also increase, leading to a further expansion of the dispersion. The contact takes on the amphipathic character of the surfactant as a result of the adsorption process. The surface tension decreases within the aqueous and lipid phase with an increase in the concentration of the surfactant. A longer period of sonication results in more fragmentation of the particles and a smaller overall size of the dispersion [24]. It can be seen from the equation that the interaction between the surfactant and the sonication had a substantial effect. Both of these factors, acting independently, had an impact on the particle size. The interplay of these two elements contributes to a smaller size distribution of the particles in the sample. In contrast to the way the lipid and surfactant interact, the lipid and the sonication both have a relatively small impact on the size reduction.

### 2.4. Effect of Independent Variables on EE

According to Supplemental Table S4, the EE of BCP-NLC dispersion was determined to be in the range of 74.58% to 86.74%. The importance of the model may be inferred from the quadratic model, which has an F-value of 55.54. In Figure 2B, you can see a 3D plot, a contour plot, and a comparison of the projected value to the actual value of the response variable entrapment efficiency. An F-value of 10.40 in relation to the pure error indicated that the Lack of Fit was a significant finding. The values that were coded for the independent variables that were chosen are shown as an equation here.

Y2 = +86.16 + 2.00A + 0.4325B + 0.3250C − 0.1225AB + 0.6475AC − 0.7275BC − 4.63 A^2^ − 4.00B^2^ − 4.54C^2^

The R^2^, the adjusted R^2^, and the predicted R^2^ value were found to be 0.9862, 0.9684, and 0.8017, respectively. The three values were discovered to be very similar to one another (the variance was lower than 0.2). The absence of a fit demonstrates that the data of the best-fitting model are comparable, which validates the accuracy of the model’s ability to account for experimental findings. The findings show that there is a correlation between an upsurge in lipid concentration and a rise in the quantity of entrapment. Due to the fact that BCP is partitioned into the lipid phase, an upsurge in the quantity of lipids leads to improved EE, which is exactly what was supposed to happen. An upsurge in the surfactant concentration was observed to some degree to be associated with an improvement in EE. It is possible that the surfactant will advance the stability of the vesicle and the encapsulation of the medication into nanocarriers [24]. An upsurge in the total amount of sonication time was shown to result in a noticeable improvement in the EE. It was anticipated that there would be an increase in entrapment as a result of the alteration of maximal lipid to nano size brought about by the separation of microparticulate particles from the BCP-NLC. This lowered the EE brought about by shortened sonication duration. An upsurge in the sonication causes the BCP-NLCs to become smaller, which in turn causes the bigger particles to be broken down into smaller particles [25]. The interaction between the surfactant and the sonication time has a significant bearing on the EE, as shown by the equation. The combination of these two elements contributes to a favorable rise in the efficiency of trapping. The effect between sonication and the lipid has a bigger influence than the effect between the surfactant and lipid. This can be seen when comparing the two types of lipid interactions. The effect between sonication and the lipid might be considered a factor that favors the rise in entrapment.

The findings were verified using a formulation that had 120 mg of lipid, 30 mg of surfactant concentration, 5 min of sonication duration, and a desirability rating of 1. Following the execution of the selected formulation, it was observed that the vesicle size was 210.86 nm with a PDI of 0.263. Both the EE and the zeta potential of the BCP-NLC came in at −26.97 mV and 86.74%, respectively. This formulation was chosen for additional characterization because of its efficacy.

### 2.5. Validation of Experimental Design

The responses obtained were compared with the new formulation. This comparison indicated an ideal formula that consisted of 120 mg of lipid concentration and 30 mg of surfactant concentration with a sonication time of 5 min. The fact that the findings of the experiments were found to be consistent with one another indicates the formulation’s validity and dependability once it was optimized.

### 2.6. Optimized BCP-NLC Vesicle Size, Zeta Potential and EE

**Figure 3:**
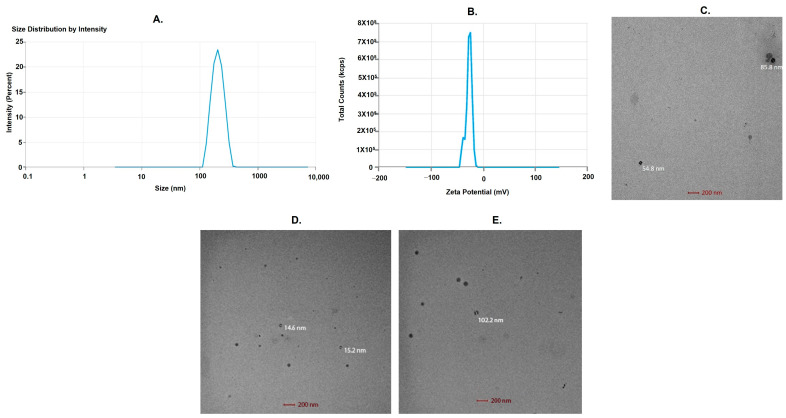
(**A**) Average particle size using zetasizer, (**B**), zeta potential, (**C**) transmission electron micrograph (TEM) of optimized BCP-NLC, (**D**) TEM of BCP-NLC formulation at low lipids/surfactants concentration, (**E**) TEM of BCP-NLC formulation at high lipids/surfactants concentration.

The vesicle size of BCP-NLCs that were optimized was determined to be 210.86 nm (Figure 3A). A zeta potential of −26.97 mV was demonstrated by the formulation that has been optimized (Figure 3B). When the zeta potential is greater than ten, it implies that the produced dispersion has stable electrostatic repulsion, which reduces the likelihood of the particles coalescing or aggregating [25]. The negative charge seen on the particles was consistent with the hypothesis that the anionic nature of the lipid was the source of the charge. The formulation’s optimization resulted in an entrapment efficiency of 86.74%. Figure 3C–E show the TEM images that illustrate the shapes of the NLCs as they appeared in the various concentrations of lipids/surfactants (optimized, low, and high, respectively), and the images also demonstrate the particle dispersion throughout the image areas.

### 2.7. Stability Testing

To assure the quality, safety, and efficacy of the drug throughout its shelf life, a stability study according to ICH requirements was conducted since it displayed superior quality features. Stability testing after 1, 3, and 6 months observed no changes in the color, odor, homogeneity, pH, and viscosity of the NLCs formulation (Table 1).

### 2.8. Drug Release Study: In Vitro

**Figure 4:**
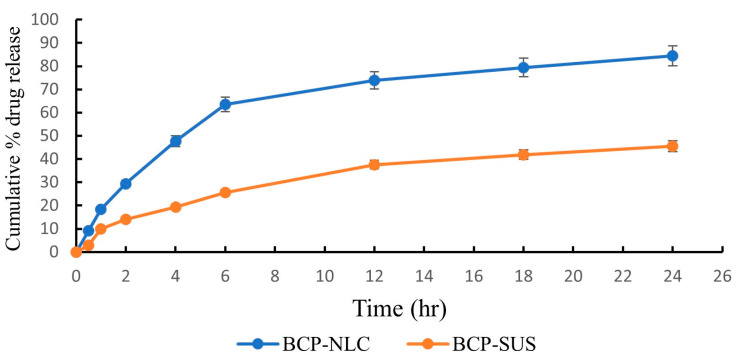
In vitro drug release from BCP-NLC and BCP-SUS at pH 7.4. The data are statistically significant with a *p*-value less than 0.0001 for both formulation and suspension.

The drug permeation study through a dialysis bag was employed to evaluate the release of BCP from both the BCP-NLC and the β-caryophyllene suspension (BCP-SUS) in pH 7.4. The main theme of this investigation was to evaluate the discharge of BCP. Although this was the case, the release of the medication from the BCP suspensions was much too slow, with only 45.48 ± 2.32% of the drug being released after 24 h when combined with drug precipitation in the dialysis bag. After 24 h, we were able to calculate that the BCP-NLC formulation had a cumulative drug release of 84.42 ± 3.21% of the medication. The data are statistically significant with a *p*-value less than 0.0001 for both formulation and suspension. At first, it seemed as though the medication might quickly discharge from the lipid surface of the BCP-NLC. However, this turned out not to be the case. It was found that there was a correlation between the two variables that was substantial enough to warrant statistical analysis. After tuning the BCP-NLC, a biphasic sort of release could be seen coming from both the BCP-NLC and the BCP suspension. Because of the lower particle size of the NLC that was generated, the BCP-NLC had a release period of 24 h (Figure 4). This may have been the effect of the smaller droplet size.

The data suggested that the release of medication from the BCP-NLC could be accounted for using the Korsmeyer–Peppas release model. This was due to the fact that this model had the highest value of the coefficient correlation (R^2^), which was calculated to be 0.9402. In addition, the drug release pattern followed Fickian diffusion with a BCP release form biphasically, which demonstrated that the drug was encapsulated within the center of lipids. The effectiveness of the BCP-NLC was proved in drug release studies by the fact that in comparison to the BCP suspensions, it displayed a maximum drug release that was 1.86 times higher than the BCP suspensions.

### 2.9. Drug Release Study: Ex Vivo

**Figure 5:**
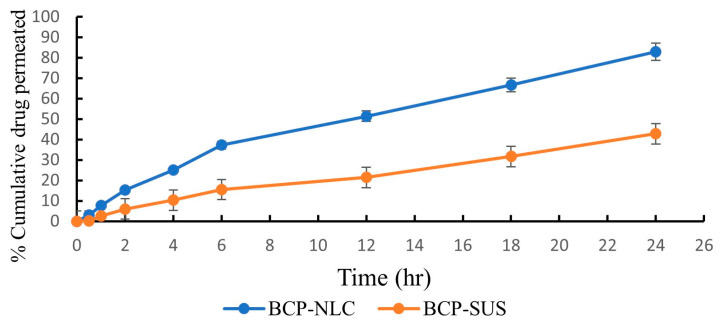
Ex vivo graphs show % cumulative drug (BCP) penetrated through the skin using BCP-NLC and BCP-SUS. The data are statistically significant with a *p*-value less than 0.0001 for both formulation and suspension.

In the ex vivo release investigation, rat skin was taken to evaluate the release and act as a barrier on the Franz diffusion cell. This allowed the researchers to determine how well the drug (BCP) was able to permeate through the barrier and into the bloodstream of the rats. As a result, we could identify which mode of distribution was the most efficient. In contrast to the BCP suspension, which showed a penetration of 42.79 ± 1.29% after 24 h, the BCP-NLC revealed a penetration of 82.90 ± 3.16% over the same time period (Figure 5). It was found that there was a correlation between the two variables that was substantial enough to warrant statistical analysis. The data are statistically significant with a *p*-value less than 0.0001 for both formulation and suspension. It was found that the sequence of release observed during the ex vivo permeation investigations was related to that which was observed during the in vitro tests. This link was made after it was noticed that the two sets of findings were connected. When the drug permeation shown by the BCP suspension was compared to the drug permeation shown by the BCP-NLC, the BCP-NLC displayed a 1.94 times increase in drug permeation. This could be due to the fact that the BCP-NLC had a longer retention period, which leads to an increased penetration rate and also to the anticipated prolonged permeation of the active component, demonstrating its efficiency in drug release assays. Alternatively, this could be due to the fact that the BCP-NLC had a higher molecular weight, which in turn led to an increased penetration rate [26].

### 2.10. Dermatokinetic Estimation

**Figure 6:**
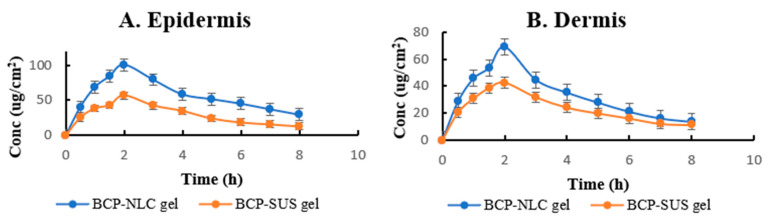
Shows topical application of BCP-NLC and BCP-SUS gel on the skin of rat (**A**) Epidermis and (**B**) Dermis. The data are statistically significant, with a *p*-value of 0.0002 for formulation and less than 0.0001 for suspension in epidermis. In dermis, the data are also statistically significant, with a *p*-value less than 0.0001 for formulation and suspension.

The dermatokinetic profile of NLC’s formulation as well as the unbound drug-loaded gel are displayed in Figure 6A and 6B, respectively. The maximum concentration (Cmax_skin_) of the BCP-NLCs-loaded gel was discovered to be 100.69 ± 2.65 μg.cm^−2^ in the epidermis and 68.84 ± 2.86 μg.cm^−2^ in the dermis. These values were confirmed to be statistically significant. The Cmax_skin_ of the BCP-SUS incorporated gel was determined to be 56.28 ± 3.16 μg.cm^−2^ for the epidermis and 42.50 ± 3.29 μg.cm^−2^ for the dermis. These values were confirmed to be statistically significant. It was discovered that the time to reach the peak concentration of drug (Tmax) of the BCP-NLCs incorporated gel was 8 h in both the epidermis and dermis, whereas the Tmax of the BCP-SUS incorporated gel was discovered to be 2 h in both the epidermis and dermis. It was discovered that the area under the curve from 0 to 8 h (AUC_0–8h_) of the BCP-NLCs incorporated gel in the epidermis was 458.94 ± 25.83 μg.cm^−2^.h and that the AUC_0–8h_ of the gel in the dermis was 267.09 ± 21.93 μg.cm^−2^.h The epidermis and dermis revealed an AUC_0–8h_ of 235.93 ± 13.21 and 186.60 ± 9.48 μg.cm^−2^.h for the BCP-SUS incorporated gel, respectively. It was discovered that the elimination rate constant (Ke) of the NLCs-loaded gel in the epidermis was 0.1014 ± 0.04 and that the Ke of the gel in the dermis was 0.1649 ± 0.06. Ke values of 0.1498 ± 0.05 and 0.1437 ± 0.03 were recorded in the epidermis and dermis, respectively, when BCP-SUS incorporated gel was applied. The data are statistically significant, with a *p*-value of 0.0002 for formulation and less than 0.0001 for drug in epidermis. The data are also statistically significant for formulation and suspension in the dermis with a *p*-value less than 0.0001. When compared to the preparation of traditional gel, the NLC-based gel displayed greater penetration and skin retention due to its higher Tmax and higher drug concentration in the skin layers. This was the case even though the conventional gel preparation used a lower dose of the active ingredient. The BCP targeted site is the skin, as the drug is intended for skin-related diseases. The observed Cmax is sufficient to deliver the drug in therapeutic concentration for topical use. The findings of this research are in agreement with the previously published article [27]. The research indicates that BCP-NLCs are likely to lodge themselves in the surface layers (epidermis), whilst the medicine is likely to permeate into the deeper layers (dermis). Because of the sticky nature of the lipid-based gel’s composition, it is more probable that a high concentration of the drug will be detected in the layers of the skin [28].

### 2.11. Antioxidant Activity by DPPH Assay Method

**Figure 7:**
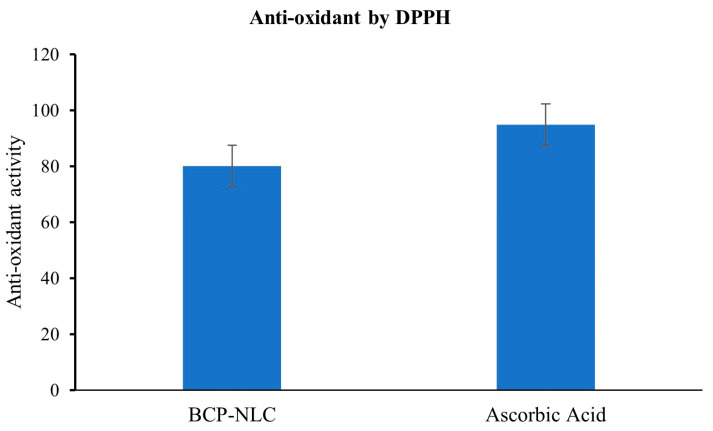
Antioxidant study by DPPH.

The ability of DPPH to reduce was determined by examining changes in color (purple to yellow), which was measured at 515 nm. According to the findings, the BCP-NLC exerted a significant amount of impact on the process of free radical scavenging. The fact that the antioxidant activity of the BCP-NLC was discovered to be stronger as a result of the combined impact of BCP is evidence that these compounds are effective as powerful antioxidants. It is hypothesized that the synergistic impact of BCP in combination with Transcutol P, which possesses significant antioxidant activity, could be the explanation for the effective antioxidant potential in the BCP-NLC [29]. The observed antioxidant activity of the BCP-NLC was 80.12%, while the antioxidant activity of ascorbic acid was determined to be 94.92%, as shown in Figure 7 [26].

### 2.12. Estimation of Depth of Permeation by CLSM

**Figure 8:**
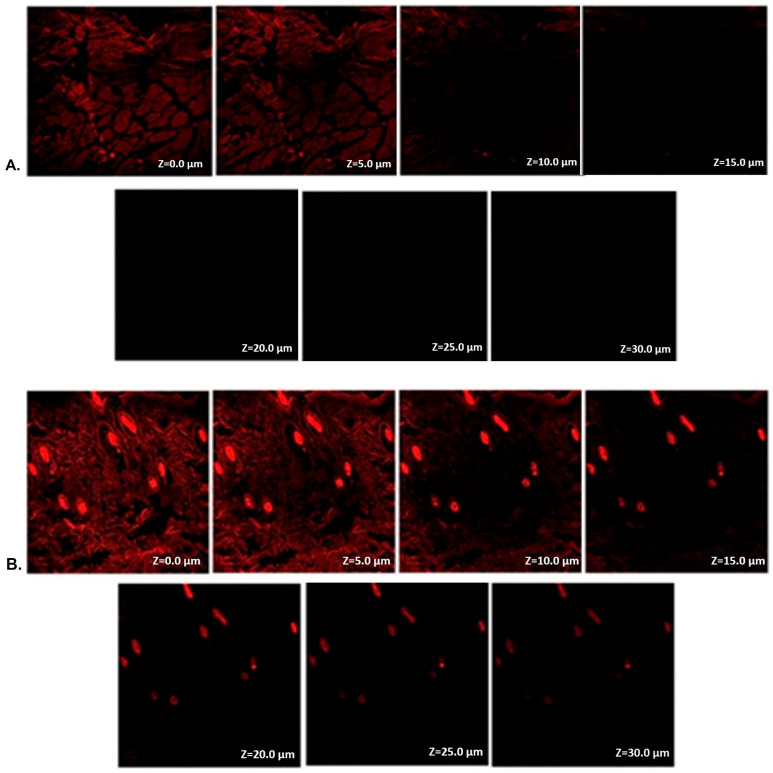
CLSM of (**A**) Rhodamine B solution with BCP suspension and (**B**) Rhodamine B incorporated BCP-NLC.

The control was a rhodamine B- hydroethanolic solution, and the improved NLC formulation was loaded with rhodamine-B dye. The rhodamine B incorporated into the BCP-NLC is shown to be stable, and no separation of phase is shown. This includes that rhodamine B is suitable for this formulation and gel. Research using CLSM was carried out to determine the degree to which the BCP-NLC and BCP suspension were able to pass through the rat skin. It was noticed that the exhibited fluorescence at its most intense level for the BCP suspensions was permeated up to a depth of 10 µm (Figure 8A). On the other hand, it was seen all the way down to a depth of 30 µm in the BCP-NLC (Figure 8B), which indicates that the BCP in the BCP-NLC has a more uniform distribution than it does in the BCP suspension. In conclusion, as compared to the BCP solution, the BCP-NLC demonstrated about three times deeper penetration in the skin. The findings of this investigation were found to be consistent with the conclusions drawn from the ex vivo permeation test.

## 3. Conclusions

Cold homogenization was used in the formulation of the NLCs-loaded BCP, and the BBD was used for the optimization. The improved penetration into the skin is due, in part, to the tiny particle size and high PDI of the modified formulation. The presence of surfactant had an effect on the tiny particle size, and the lower HLB value of the lipid increased the entrapment efficiency. The release was kept under control for a period of twenty-four hours by the improved formulation. Both penetration and skin retention are significantly enhanced with the NLCs-loaded gel. Based on the results that were obtained, it was determined that beta-caryophyllene-loaded NLCs had the potential to be studied for topical distribution with increased skin retention. The newly formulated preparation was examined for its size, EE, and zeta potential after being improved using the BBD. The formulation that was chosen underwent tests to determine its ex vivo skin retention, dermatokinetics, in vitro release, antioxidant and CLSM study. The findings of the characterization of the NLCs demonstrated that the particles had a spherical form and a size of 210.86 nm (0.263 PDI). The entrapment efficiency was determined and found to be good—that is, 86.74%, and the zeta potential was measured to be −26.97 mV. The in vitro release investigation showed that NLCs were capable of releasing regulated amounts of beta-caryophyllene for up to 24 hrs. In the evaluation of the traditional gel formulation, the ex vivo investigation demonstrated a 1.94-fold increase in the skin’s capacity to retain the substance. According to the findings of the study, NLCs loaded with beta-caryophyllene have the potential to be investigated for use as a topical administration method in skin disorders with enhanced skin retention and effectiveness.

## 4. Materials and Methods

### 4.1. Materials

β-caryophyllene, DPPH, rhodamine and a dialysis bag were purchased from Sigma-Aldrich Co. Compritol 888ATO, Precirol ATO5, Apifil, glycerol monostearate (GSM) and Transcutol P were purchased from Gattefosse India Pvt Ltd. (Mumbai, India). Stearic acid, Tween-80, Tween-40, Tween-20, Span-20, Span-60, Span-80, castor oil, olive oil, oleic acid, Carbopol 940, linseed oil and propylene glycol were purchased from Mumbai, India- Research Lab Fine Chem Industries. Methanol was purchased from Thomas Baker Chemicals Pvt Ltd. Mumbai, India.

### 4.2. Methods

#### 4.2.1. Preliminary Screening of Solid Lipids

When determining which lipid to use, the extent to which a drug could dissolve in the solid lipid was a factor that was taken into account. Following the placement of a quantity of distinct solid lipids into individual vials, those vials were subjected to heating on a magnetic stirrer at a temperature of 10 ± 1 °C. This temperature was somewhat greater than the threshold at which the solid lipid would begin to be melting. After that, BCP was added in a methodical manner until the saturation point was achieved, which was evaluated by visually evaluating the mixture. Once this point was reached, the mixture was considered to be saturated. After that, the mixture was given a vortex for 5 min before being placed into an iso-thermal shaker for around 48 h at 25 ± 0.5 °C. After completing the centrifugation for 13 min at 3000 rpm, the supernatant that was produced was afterward taken, diluted with methanol, and evaluated by UV spectroscopy [30,31,32].

#### 4.2.2. Preliminary Screening of Liquid Lipids and Surfactants

The degree to which BCP is soluble in a variety of liquid lipids and surfactants was evaluated by placing a sufficient volume of BCP in a glass vial containing 5 mL of each kind of liquid lipid or surfactant. The vials were swirled using a magnetic stirrer at a rate of 50 revolutions per minute for a period of twenty-four hours at a temperature of 37 °C ± 1.0 °C. The vial was centrifuged for a total of 30 min at a speed of 1500 rpm. Using vacuum filtration, the materials were filtered through a membrane filter of 0.45 μm. In order to calculate the BCP, the supernatant was first appropriately diluted with methanol, and then, it was examined using an ultraviolet (UV) spectrophotometer (UV-1601, Shimadzu, Japan) at 205 nm [33].

#### 4.2.3. Optimization of BCP-Loaded NLC (BCP-NLC)

The 3-factor, 3 levels BBD (software Design Expert v13) was utilized for BCP-loaded NLCs formulation. As per the BBD, 17 runs of BCP-loaded NLCs formulations were formulated and investigated. The formulation is going to be perfected by optimizing it according to numerical validation and the desirability of the parameters that were acquired from the design of the experiment [34,35]. It was determined that the lipid phase, the surfactant, and the sonication period each acted as independent factors. On the other hand, the vesicle size and the % EE were taken into consideration as dependent variables (Supplementary Table S5).

#### 4.2.4. Preparation of NLCs

With the selected lipids (Compritol 888ATO and Linseed oil) in the ratio of 1:1 and surfactant (Tween 80), BCP-NLCs were formulated using the cold homogenization technique. In a nutshell, linseed oil and Compritol 888ATO were fused together. BCP, a surfactant, and a permeation enhancer called Transcutol P were mixed into the molten lipid mass and stirred at a predetermined amount of time using a Probe Sonicator (Sonapros PR-250, Oscar Ultrasonics, Mumbai, India). Both stages were kept at the same temperature throughout (70 °C). The combination was cooled quickly by using an ice bath. Using a mortar and pestle that had been thoroughly cleaned and dried, the BCP that contained the solid lipid mass was pulverized into NLCs. After that, the micro-particles were mixed into an aqueous solution of propylene glycol (2% *v*/*v*) at a temperature of 4 °C [36,37].

### 4.3. Characterization of NLCs

#### 4.3.1. Particle Size, Zeta Potential and Morphology

The zetasizer was used to determine the size, PDI, and zeta potential of the optimized BCP-NLCs formulation. Before conducting the particle size evaluation by utilizing dynamic light scattering, the generated NLCs dispersion was first diluted with 10 times its original volume of Milli-Q water. The measurement was then carried out at a temperature of 25 °C, and electrophoretic mobility was used to determine the value of the zeta potential. Transmission electron microscopy (TEM) was utilized in order to analyze the morphological pictures of the produced dispersion [38].

#### 4.3.2. Entrapment Efficiency

The direct technique was used to estimate the %EE of BCP-NLCs in the dispersion [39,40]. After the prepared formulation had been centrifuged for five minutes at a speed of 5000 rpm, the lipid carrier dispersion in the supernatant was put through ultracentrifugation at a speed of 35,000 RPM. After removing the supernatant, the settling nanoparticles were centrifuged to separate the solid lipid and then subjected to lysis and extraction with acetonitrile. The following equation was utilized in order to obtain the %EE. The resulting solution was passed through a filter before being examined using an ultraviolet (UV) spectrophotometer (UV-1601, Shimadzu, Japan) at 205 nm [41,42,43].

$$\%\mathrm{EE}=\frac{\mathrm{Amount}\;\mathrm{of}\;\mathrm{drug}\;\mathrm{in}\;\mathrm{lipid}\;\mathrm{nanocarriers}}{\mathrm{Total}\;\mathrm{amount}\;\mathrm{of}\;\mathrm{drug}\;\mathrm{taken}}\times 100$$

#### 4.3.3. Stability Studies

The major objective of stability analyzing is to ascertain how the amount of the drug shifts over the course of time in response to variation in temperature and RH. In accordance with the suggestions provided by the ICH, the BCP-NLC formulation underwent stability research that lasted for a full six months inside a stability chamber. The NLCs were kept in stability chambers separately at 25 °C ± 2 °C/60% RH ± 5% RH, 32 °C ± 2 °C/60% ± RH 5% RH, and 40 °C ± 2 °C/75% RH ± 5% RH. The initial, first-month, second-month, and third-month samples as well as sixth-month samples were collected and analyzed for differences in color, odor, homogeneity, pH, and viscosity [44].

#### 4.3.4. In Vitro Drug Permeation Study of NLCs Dispersion

The dialysis bag method was utilized in order to understand the NLCs dispersion with respect to the in vitro drug release profile [44]. With a molecular weight cutoff of 120,000 Da, the medication solution and the NLCs dispersion were each hermetically sealed in their own compartment of the dialysis bag. The USP monograph served as the basis for the decision to use 15 milliliters of buffered solution with 0.15% sodium lauryl sulfate as the medium in which to submerge the dialysis bag. Before it was used, the buffer was sonicated to remove any foam and air bubbles that could have been present. To keep the sink condition constant, an aliquot of 1 milliliter was taken out at predetermined intervals, and an equivalent volume of new release medium was added at those intervals. The HPLC technique was used to conduct the analysis of the samples. The acquired data were evaluated by inserting the detected values into several equations, and a best-fit release model was discovered by utilizing the “DD Solver” add-in for Excel [41,45].

#### 4.3.5. Preparation of BCP-NLC Gel

Carbopol 940, which had a weight-to-weight ratio of 1%, was first passed on a sieve (size: #60) in order to prevent cluster and lump formation before it was dispersed in purified water to make a gel. Using a magnetic stirrer, the mixture was stirred for ten minutes at a speed of 1500 revolutions per minute, and then the pH was immediately adjusted to 7.4 using triethanolamine. After allowing the gels to equilibrate for another twenty-four hours at room temperature, nanoscale liquid crystals were then disseminated throughout the gel. The gel was left out at room temperature overnight to allow any trapped air to escape [46]. For the creation of the pure BCP-loaded gel, a technique quite identical to the previous one was followed.

#### 4.3.6. Ex Vivo Skin Permeation Studies

The skin permeation experiments and dermatokinetics on rodent skin were properly approved by the Standing Committee of Bioethics Research at Prince Sattam bin Abdulaziz University in Al-Kharj, Saudi Arabia (SCBR-020-2023). In order to conduct ex vivo permeation investigations on male Wistar rat skin, the NLCs-embedded gel formulation was tested and assessed. Fresh rat skin was collected, and a hair clipper was used to shave the dorsal portion of the skin. The epidermis of the skin was positioned such that it faced the donor while it was placed between the donor compartment and the receptor compartment of Franz diffusion cells (0.785 cm^2^). The contact surface area of the Franz diffusion cells was 0.785 cm^2^, and the receptor compartment had a capacity of 10 mL. It was filled with phosphate-buffered saline with a pH of 7.4. Placing the gel preparation on the donor part, it was spread across the epidermis. The setup was kept at 32 ± 1 °C, with a continuous stirring speed of 200 revolutions per minute. Following the collection of the sample aliquots at the predetermined time intervals (0, 0.5, 1, 2, 4, 6, 12, 18, and 24 h), an equal volume of fresh medium was substituted for the volume that was removed. A technique of analysis was utilized in order to estimate the amount of medicine that was able to pass through [47,48,49].

#### 4.3.7. Dermatokinetics

The skin of a healthy Wistar rat was removed for the dermatokinetic investigation. This approval included both the methodology and the execution of the investigations. The dermatokinetic investigation was conducted at various time intervals in both the epidermis and the dermis in order to establish the optimal formulation of NLCs. It is necessary to estimate the drug’s retention in the various layers of skin as well as its removal or breakdown by enzymes in the skin. The highest concentration of the drug that can be reached in the skin layer is attained by the application of a high concentration of the drug. The concentration of the medication plays a role in how well it is absorbed via the skin. The kinetic characteristics of the medications that are administered topically are changed as a result of these factors. Studies of the dermatokinetics of BCP-NLCs gel and BCP-SUS gel were conducted at certain time-point using fresh rat skin as per the method’s ex vivo skin permeation study. After the experiment was finished, the skin was washed with the buffer to clean any excess amount of the drug that had not been absorbed, and it was then separated into the epidermis and dermis layers. Acetonitrile was used in order to extract the medication from the various layers of peeled-off skin. After centrifuging the solution that had been used to extract the medication, the supernatant was recovered. The samples that were obtained were put through an analysis with an HPLC technique after being filtered through a nylon filter of 0.2 μm. Plots were made using the estimated levels of BCP concentration in the epidermis and dermis layers of the skin, and the dermatokinetics was analyzed. For the purpose of determining Tmax, Cmax skin, AUC0-8h, and Ke, we used the non-compartmental pharmacokinetic model [50,51].

### 4.4. Antioxidant Activity

#### 4.4.1. DPPH Assay

DPPH (5,5′-Dithiobis-2-nitrobenzoic acid) is a radical that is known to be stable, and when its spare electron or hydrogen radical is delocalized, it creates a color that is described as being very dark violet. In order to evaluate the antioxidant capacity of the BCP-NLC, it was compared with the standard reference, i.e., ascorbic acid standard and BCP suspension. The investigation was carried out to estimate the capacity of the DPPH solution to scavenge free radicals at ambient temperature. Methanolic solutions with varying concentrations of all three materials, ranging from 1 to 80 µg/mL, were prepared. One milliliter of each sample was then diluted with one milliliter of DPPH methanolic solution. After waiting for 30 min, the combination was scanned while keeping 95% methanol as a blank at 515 nm [52]. The below eq. was utilized in order to compute the % DPPH inhibition achieved:

$$\%\;\mathrm{Inibition}\;\mathrm{of}\;\mathrm{DPPH}\;\mathrm{Radical}=\frac{\mathrm{Ac}-\mathrm{As}}{\mathrm{Ac}}\times 100$$

where Ac is the absorbance of the control and As is the absorbance of the sample

#### 4.4.2. Estimation of Penetration Depth by CLSM Technique

CLSM research was used to provide an estimate of the prepared NLC’s penetration depth. The control was a rhodamine-B hydroethanolic solution, and the improved NLC formulation was loaded with rhodamine-B dye. The penetration depth in rat skin was measured by CLSM, and the results were compared to the control. “Franz diffusion cells” were used to evaluate the in vitro skin penetration of rhodamine B-loaded BCP-NLC gel and rhodamine-B hydroethanolic solution over a period of eight hours as part of the CLSM experiment. After preparing glass slides of skin samples, the samples were viewed using a CLS microscope, and the penetration depth of the probe dye was evaluated [53].

#### 4.4.3. Statistical Analysis

The outcomes of the studies were analyzed by Box–Behnken Design (Design Expert: v13). The studies were carried out three times, and the results were expressed as the mean and standard deviation (SD) using one-way ANOVA analysis by minitab v18.

## Figures and Tables

**Table 1:** Stability testing data.

Test	25 °C ± 2 °C/60% RH ± 5% RH			32 °C ± 2 °C/60% ± RH 5% RH			40 °C ± 2 °C/75% RH ± 5% RH		
	1	3	6	1	3	6	1	3	6
Color	Clear	Clear	Clear	Clear	Clear	Clear	Clear	Clear	Clear
Homogeneity	Good	Good	Good	Good	Good	Good	Good	Good	Good
pH	6.7	6.8	6.8	6.8	6.8	6.8	6.9	6.9	6.8
Viscosity	Good	Good	Good	Good	Good	Good	Good	Good	Good

## Data Availability

Not applicable.

## Supplementary Materials

The following supporting information can be downloaded at: https://www.mdpi.com/article/10.3390/gels9070550/s1. Figure S1. Solubilization of BCP in various Solid lipids. The data is statistically significant with *p*-value less than 0.005. Table S1. Solubility of BCP in Solid lipids. Table S2. Solubilization of BCP in liquid lipids. Table S3. Particle size and PDI of nanoparticles with different surfactant. Table S4. Observed responses in BBD software for the optimization of BCP-NLC formulation and summary of results of regression analysis for responses Y1 and Y2 for fitting to quadratic model. Table S5. Box Behnken Design (BBD) independent and dependent variables for the development and optimization of BCP-NLC.
