# Supplementary material for: Beta Caryophyllene-Loaded Nanostructured Lipid Carriers for Topical Management of Skin Disorders: Statistical Optimization, In Vitro and Dermatokinetic Evaluation
Source: Gels. 2023 Jul 6;9(7):550. doi: 10.3390/gels9070550 (PMC10378941; doi:10.3390/gels9070550)
Supplement: Supplementary file 1 [file gels-09-00550-s001.zip › gels-2447742-supplementary.pdf]

gels

Article

Beta Caryophyllene-Loaded Nanostructured Lipid Carriers for Topical Management of Skin Disorders: Statistical Optimization, In Vitro and Dermatokinetic Evaluation

Mohammed Ghazwani [1,*], Umme Hani [1], Mohammed H. Alqarni [2] and Aftab Alam [2]

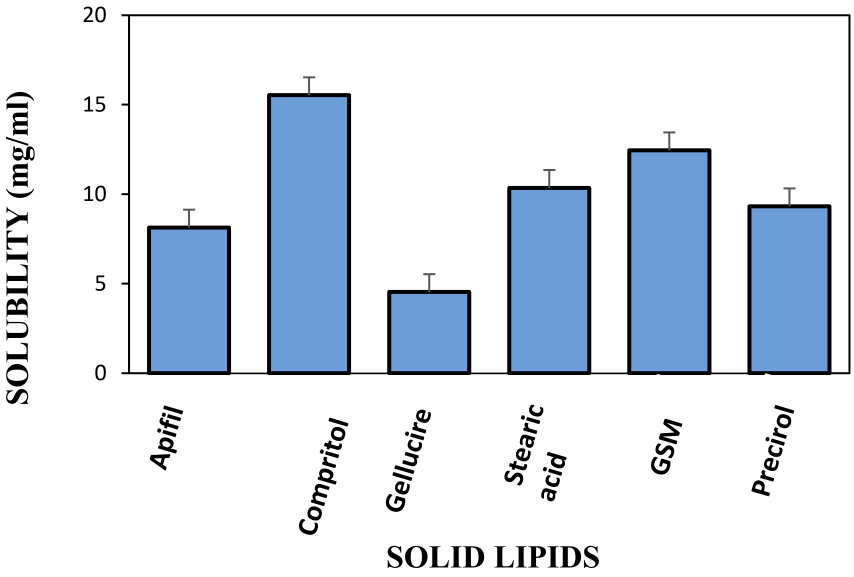

Figure S1. Solubilization of BCP in various Solid lipids. The data is statistically significant with P-value less than 0.005.

Table S1. Solubility of BCP in Solid lipids.

Code	Components	Solubility (mg/ml)
A1	Apifil	8.13 ± 1.45
A2	Compritol 888ATO	15.53 ± 2.26
A3	Gellucire	4.53 ± 0.73
A4	Stearic acid	10.35 ± 2.01
A5	GSM	12.45 ± 2.84
A5	Precirol ATO5	9.32 ± 1.21

Table S2. Solubilization of BCP in liquid lipids.

Code	Components	Solubility (mg/ml)
C1	Oleic acid	12.74 ± 1.32
C2	Castor oil	7.12 ± 0.83
C3	Linseed oil	14.89 ± 1.52
C4	Olive oil	5.27 ± 0.38
C5	Soya Bean oil	6.63 ± 0.62

Table S3. Particle size and PDI of nanoparticles with different surfactant.

Batches	Surfactant	Particle size (nm)	PDI
F1	Tween 20	243 ± 12	0.329 ± 0.13
F2	Tween 60	238 ± 10	0.311 ± 0.11
F3	Tween 80	210 ± 8	0.263 ± 0.08
F4	Span 20	285 ± 14	0.653 ± 0.23
F5	Span 60	279 ± 12	0.621 ± 0.26
F6	Span 80	289 ± 13	0.553 ± 0.18

Table S4. Observed responses in BBD software for the optimization of BCP-NLC formulation and summary of results of regression analysis for responses Y1 and Y2 for fitting to quadratic model.

Runs	Independent Variables			Dependent Variables	
	A	B	C	Y_1	Y_2
1	150	30	4	253.41	78.13
2	120	40	6	216.56	78.16
3	90	40	5	239.48	75.68
4	120	20	6	220.14	77.47
5	150	20	5	260.23	79.64
6	120	30	5	210.86	86.74
7	90	30	6	232.14	74.58
8	150	30	6	246.85	80.35
9	120	30	5	211.52	86.25
10	120	30	5	212.14	86.01
11	90	30	4	238.84	74.95
12	90	20	5	241.31	75.85
13	120	30	5	212.54	85.87
14	150	40	5	251.65	78.98
15	120	30	5	211.33	85.95
16	120	20	4	215.65	75.64
17	120	40	4	213.52	79.24
Quadratic model	R^2	*Adjusted* R^2	*Predicted* R^2	*SD*	*C.V. %*
Response (Y_1)	0.9880	0.9726	0.8135	2.90	1.27
Response (Y_2)	0.9862	0.9684	0.8017	0.79	0.99

A= Lipid concentration (mg), B= Surfactant concentration (mg), C= Sonication Time, Y1= Particle size (nm), Y2= Entrapment efficient (%)

Table S5. Box Behnken Design (BBD) independent and dependent variables for the development and optimization of BCP-NLC.

Variables	Levels Used		
	Low (-1)	Medium (0)	High (+1)
Independent Variables			
A= Lipid Concentration (mg)	90	120	150
B= Surfactant Concentration (mg)	20	30	40
C= Sonication Time (Min)	4	5	6
Dependent Variables			
Y_1 = Particle size (nm)	Minimum		
Y_2 = Entrapment efficient (%)	Maximum		
